# Author Correction: A nascent riboswitch helix orchestrates robust transcriptional regulation through signal integration

**DOI:** 10.1038/s41467-025-56683-3

**Published:** 2025-02-28

**Authors:** Adrien Chauvier, Shiba S. Dandpat, Rosa Romero, Nils G. Walter

**Affiliations:** 1 Single Molecule Analysis Group and Center for RNA Biomedicine, Department of Chemistry, University of Michigan, Ann Arbor, MI USA; 2 Present Address: Intel Corporation, Hillsboro, OR USA

**Keywords:** RNA, Single-molecule biophysics, Riboswitches

Correction to: *Nature Communications* 10.1038/s41467-024-48409-8, published online 10 May 2024

In the version of the article initially published, the NSF grant MCB 2140320 was missing from the Acknowledgements section and has now been added to the HTML and PDF versions of the article.

